# 

**Published:** 1832-08

**Authors:** 


### MONTHLY LIST OF MEDICAL BOOKS.

[*Medical Works cannot be entered on this List unless a copy be sent for the purpose, the titles of Books having frequently been sent to us as published which have not been published for weeks, or even months, after.*]

The Cyclopædia of Practical Medicine. Edited by JOHN FORBES, M.D., A. TWEEDIE, M.D., and JOHN CONOLLY, M.D. Part VII. (published in Monthly Parts,) July 1832.—Royal 8vo. Sherwood, Gilbert, and Piper; and Baldwin and Cradock, London.

At the commencement of such undertakings, it is impossible to fix precisely the limits to which the work may extend, but the editors now promise with confidence, that the Cyclopædia of Practical Medicine will be completed in about twenty parts, forming three handsome volumes, each containing as much matter as is found in an equal number of pages of the large quarto Encyclopædias.

An Introduction to the Practice of Midwifery; by the late THOMAS DENMAN, M.D., Licentiate in Midwifery of the College of Physicians, London, and Honorary Member of the Royal Medical Society at Edinburgh. Seventh Edition: with a Biographical Sketch of the Author; with additional Modern Information on the various subjects treated of in the Work, and a Dissertation on the Transfusion of Blood in the more dangerous Varieties of Uterine Hemorrhage. By CHARLES WALLER, M.D., Consulting Accoucheur to the London and Southwark Midwifery Institution; Lecturer on Midwifery, and Diseases of Women and Children, &c.—8vo. pp. 525. Cox, and Burgess and Hill, London.

The Effects of Arts, Trades, and Professions, and of Civic States and Habits of Living, on Health and Longevity: with Suggestions for the Removal of many of the Agents which produce Disease, and shorten the Duration of Life. By C. TURNER THACKRAH, Esq. Second Edition, greatly enlarged.—8vo. pp. 328. Longman and Co., London; Baines and Newsome, Leeds.

Transactions of the Medical and Physical Society of Calcutta. Vol. V.—8vo. pp 449. Calcutta, 1831. Sold by Parbury, Allen, and Co., London.

An Essay on the Periodical Discharge of the Human Female, with new Views of its Nature, Causes, and Influence on Disease: to which are added, Directions for its Management in the different Stages of Life. By JOHN POWER, M.D. &c.—8vo. pp. 70. Burgess and Hill.

A Medical Guide for the Use of Families, or Journal of Popular Medicine; explaining the Nature, Causes, and Prevention of Diseases, the immediate Management of Accidents, and the Means of Preserving Health. By the late CHARLES THOMAS HADEN, Surgeon to the Chelsea and Brompton Dispensary, &c. Second Edition, with additional Essays on the Diseases of Children.—8vo. pp. 334. Burgess and Hill, London.

This work contains an abundance of practical information, with which the managers of families should make themselves acquainted; and it is also well worth the attention of medical students and practitioners.

---

### APOTHECARIES' HALL.

NAMES OF GENTLEMEN to whom the Court of Examiners have granted Certificates:

JULY 5.

Alfred Bosworth.
Francis Jones Bradshaw, of Portsmouth
William Henry Cooke, Langley, Devon
Alfred Daniell, Colchester
Massey R. Freeman Franco, Warwick
Robert Samuel Sims, West Bergholt.

JULY 12.

Robert Bates, of Tanworth, Warwickshire
Samuel Booth, Huddersfield
Frederick Jacob Clarke, Windsor
William Gimson Willoughby, Waterless
Charles George Hewson, Topsham, Devon.
Charles Nunnely, Market Harbro'
Charles Vie Ridout, Sherborne
Mozes Rogern, Longtown, Hereford.
William Smith, Whitehaven

JULY 19.

Edward Enfield Barrow, of Norwich
Henry Burnard, Crewkerne
John Edgar Beckensgale, Salisbury
Thomas Golding Cocke, Fordham
William Arthur Harburt.
William Henry Jenner, Berkley
Thomas Haigh Martin, Holinforth
Arthur Hutton Rowlandson.
Thomas Radford.
John Stevenson, Durham.

JULY 26.

Samuel Abraham, of Teignmouth
George Atkinson, Sunderland
William Balten, Oswestry
Edward Connelly, Bromboro', Cheshire
William Gerald Dickinson, London
Abraham Evans, Exeter
James Kenworthy, Manchester
Thomas Darke Martyn, St. Columb
Septamus Rodick, Pelmarsh.

### TO CORRESPONDENTS.

A Friend. We have made up our minds as to the course we shall pursue; and the "facts" (?) mentioned are too unimportant to alter the determination we have very deliberately resolved upon.

We cannot insert the anonymous review of "Medicus:" we protest we doubt his impartiality, in spite of his anxious endeavours to make us believe it by a threefold assurance. The Analysis will be acceptable.

We differ entirely from "A General Practitioner:" we hold ourselves to be perfectly at liberty to pass a general expression of disapprobation upon any work that does not appear to us to possess sufficient merit to justify a formal review. As our own characters, and the character of our Journal, are at stake, it is not likely that we shall abuse the privilege which we, in common with other journalists, assume to belong to us.

A Student will find the subject referred to by Dr. Elliotson in the "Lancet" for July 28th. Dr. E. states, "it is a remarkable fact that Dr. Prout, who has long been weighing the atmosphere twice a day, found it suddenly become very heavy exactly at the time that cholera broke out, as if something were added to the air; and that it long continued so heavy, up to the present time." The reporter to the "Lancet" remarks in a note, that "Dr. Prout discovered the sudden increase of weight in the atmosphere, alluded to in Dr. E.'s lecture, to occur almost *on the very day* on which cholera broke out in London."

Communications have been received from Mr. A. Taylor, &c.

